# Axelrod's Metanorm Games on Networks

**DOI:** 10.1371/journal.pone.0020474

**Published:** 2011-05-31

**Authors:** José M. Galán, Maciej M. Łatek, Seyed M. Mussavi Rizi

**Affiliations:** 1 Área de Organización de Empresas, Departamento de Ingeniería Civil, Universidad de Burgos, Burgos, Spain; 2 Social Systems Engineering Centre INSISOC, Valladolid, Spain; 3 Department of Computational Social Science, George Mason University, Fairfax, Virginia, United States of America; University of Maribor, Slovenia

## Abstract

Metanorms is a mechanism proposed to promote cooperation in social dilemmas. Recent experimental results show that network structures that underlie social interactions influence the emergence of norms that promote cooperation. We generalize Axelrod's analysis of metanorms dynamics to interactions unfolding on networks through simulation and mathematical modeling. Network topology strongly influences the effectiveness of the metanorms mechanism in establishing cooperation. In particular, we find that average degree, clustering coefficient and the average number of triplets per node play key roles in sustaining or collapsing cooperation.

## Introduction

A social dilemma is a situation where the interest of the individual conflicts with the preference of the collective [1]. Each person entangled in a social dilemma has rational arguments to follow a behavior that in the aggregate leads to unfavorable outcomes for the collective. Social dilemmas are found in diverse contexts. For example, economic social dilemmas include problems associated with the provision of public goods such as national security, public health and environmental protection, where individuals can make investments into a common pool to provide a costly, non-excludable asset that benefits all regardless of how much they contribute to creating it [2]–[5]. Such “collective action” problems [6]–[8] are not limited to human social behavior. Biology abounds with examples of social dilemmas. Foraging yeast cells secrete enzymes to lyse their environment, producing a valuable common good that can be used by other cells [9]; groups of meerkats take turns as sentinels and give eventual alarm calls to the group [10]. Given the relevance of a large number of situations that correspond to the definition of social dilemma, the scientific community has expended significant capital to model and propose solutions to social dilemmas.

In the most common formalization, social dilemmas are modeled as games in which players follow different strategies. Social dilemma games are characterized by the presence of at least one deficient equilibrium: a situation that is an equilibrium, so no player has incentives to change his behavior individually, but it is not Pareto optimal, because there exists at least another possible outcome that every player prefers to the current one. Often the strategy that is collectively preferred is considered cooperative; therefore cooperators provide a benefit to the group at some cost while defectors exploit the group by reaping the benefits without bearing the costs of cooperation.

**Figure 1:**
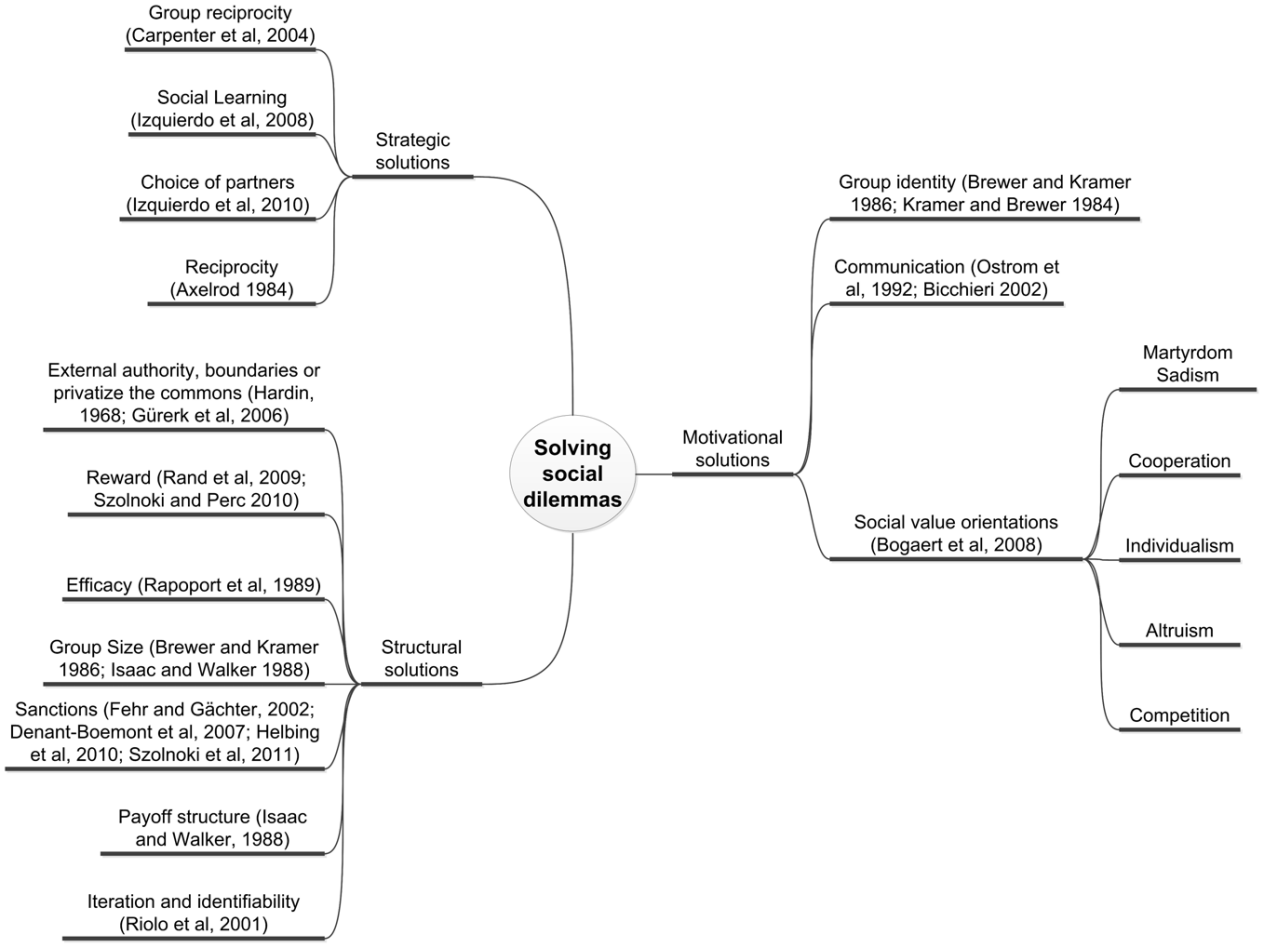
Examples of methods of solving social dilemmas based on Kollock's ontology [11]. Solutions to social dilemmas can be classified as motivational, strategic or structural depending on whether players are assumed egoist and whether the rules of the game can be changed.

Proposed methods of avoiding the generally undesirable outcomes of social dilemmas vary widely and frequently depend on context. Kollock [11] classifies these methods based on whether players are assumed egoist and whether they can change the rules of the game. His classification divides solutions to social dilemmas into motivational [12]–[16], strategic [17]–[20] or structural [2], [12], [21]–[32] (See Figure 1). In motivational solutions like moral persuasion, a player gives some weight to the results other players obtain. In strategic solutions such as reciprocity, conditional association and grim triggers, an egoistic player influences other players' behavior by expanding the range of strategies he considers. Neither solution requires coordinated or top-down modifications of the rules of the game. In structural solutions such as sanctions, central authority or privatization, the rules of the game are changed to solve the dilemma.

A mixed structural-strategic solution proposed to obtain collectively rational outcomes in social dilemmas is a sanction system in which each player can punish other players that deviate from cooperation. This type of self-imposed norm has the crucial advantage of giving the players the opportunity to sanction norm deviants selectively [33]. Behaviorally, “a norm exists in a given social setting to the extent that individuals usually act in a certain way and are often punished when seen not to be acting in this way” [34]. This notion of norms is based on social norm as opposed to legal norms, moral norms, private norms, habits or fads [35]. [36], [37] contain more extensive reviews of the meaning of social norms, and [38], [39] discuss the sociological and economic foundations of norms while [40] couches social norms in evolutionary game theory. Nevertheless, this mechanism to promote cooperation can be riddled with difficulties if punishment is costly. The punisher usually assumes the cost of promoting punishment or vigilance. This punishment cost instigates a second order “instrumental dilemma” in which players have incentives to not punish, hence causing the solution to the first “elemental dilemma” to collapse [41].

Second-order norm deviance has been studied experimentally [41]. For instance, in a controlled experiment subjects were given the option to learn of others' contributions to a public good before deciding to punish them. This mechanism mitigated the free-rider problem to some extent [3], but it created other problems like punishing high contributors [42]. A plethora of other ideas have been suggested as solutions. The threat of expulsion or ostracism seems to improve the cooperation in providing public goods [43]. Costly signaling, may result in advantageous alliances, since cooperation constitutes an honest signal of the member's quality as a coalition partner or competitor [44], [45]. Hypocritical cooperation, that is, defecting at the first level while urging others to cooperate through participation in the sanctioning system, creates more robust second-order cooperation [46]. Conformism as a psychological bias toward copying the majority can also help to stabilize cooperation [47]. group selection mechanisms, competition at two different levels, within groups and between groups [48], [49] or indirect reciprocity, the idea that good reputation will be rewarded by others, [50] have also proven to promote cooperative behavior.

The insufficiency of selective punishment as a condition to promote cooperation in social dilemmas prompted Axelrod to propose metanorms, that is, norms about how individuals follow other norms, as a mechanism to support collective cooperation in social dilemmas in evolutionary contexts [34]. Although controversial [51], [52], metanorms are touted as a mechanism for sustainable cooperative strategies in which players adhere to norms, punish defectors, and punish those who do not punish defectors [33], [38] . Mathematical analysis coupled with extensive simulation has shown that metanorms can induce both collectively and individually rational stable equilibria and that the efficiency of metanorms as a solution to social dilemmas depends on the structure of the payoff matrix. Incentives for not following norms can counterintuitively enhance the preservation of the cooperative solution; decay in punishment can cause the norm to collapse; and the details of the evolutionary algorithm, for example more explorative strategies denoted by higher levels of mutation noise, can help to preserve the norm [53]. These results suggest that metanorms as a solution to social dilemmas cannot be considered universal, because the context of the specific problem can influence its efficacy.

All theoretical research on metanorms conducted so far has assumed an evolutionary game played on a global interaction network where every player interacts with all other players. However, a more realistic view of social interactions entails embedding players in social networks that differ markedly from a completely connected interaction graph [54]. Network structures that underlie social interactions affect outcomes of such interactions; therefore shape solutions to social dilemmas. Some experimental research has sought to account for the effect of network structure on social dilemmas [55], [56]. Furthermore, experimental works on dynamics of norm enforcement and metanorms suggest that characteristics of social relations, especially interdependence, influence the emergence of norms significantly [57]–[61]. This finding implies that we may overlook relevant aspects of the problem by focusing on the direct consequences of sanctioning norm deviance without accounting for the properties of social relations over which norms and metanorms are defined. Consequently, understanding the role of network structures is essential for explaining norm enforcement.

Given that different topologies or structures of social networks can influence outcomes of social interactions [54], we adapt the metanorms game to arbitrary interaction networks and analyze the influence of network topology on the emergence of cooperation through mathematical analysis and computer simulation This hybrid methodological approach has proven to be useful in analyzing complex social models [62], [63] and extends the growing literature on games on networks [64]–[77] that is currently evolving from stylized network structures to more general interaction topologies.

The paper is organized as follows. First, we extend the metanorms game to play on networks. We then examine the dynamics and stability of a simplified version of the metanorms game mathematically. Next, we present simulation results to confirm some of the conclusions obtained analytically. Finally we present the conclusions of the work.

## Methods

### Metanorms Games on Networks

**Figure 2:**
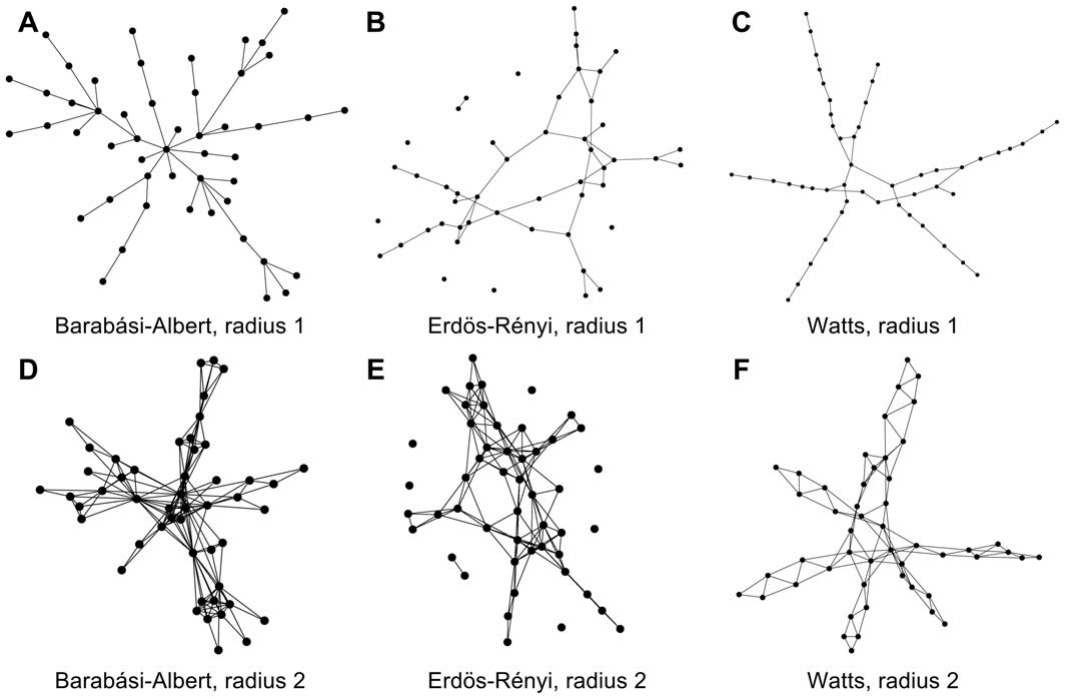
Examples of network topologies obtained with the network generation algorithms. Six sample networks with *N* = 50 and 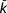 = 2. For Watts' small world network, rewiring probability β was set to 0.2. Subfigures A—C on the upper panel represent networks with radius 1. Subfigures D—E on the lower panel have neighborhoods expanded to radius 2.

We set up the metanorms game on networks by embedding 50 agents on a network developed by a network generation algorithm. We use 50 agents instead of 20 in Axelrod's default setting to make higher-order network statistics more interpretable. We used the Barabási-Albert algorithm to generate networks with discrete Pareto degree distributions [78], the Watts algorithm [79] with different values of rewiring probability (β) that smoothly interpolates between extreme cases of a regular lattice and a random network, traversing “small world” networks [80] along the way, and the Erdös-Rényi random networks [55]. A link between two agents represents an opportunity for direct interaction between them. A set of all direct links to an agent is the neighborhood of the agent. To explore the effect of clustering in the networks more clearly, we have also considered agents with a distance or radius of two where radius is defined as the minimum number of edges that it takes to link one agent to another (See Figure 2).

Once agents are embedded on the underlying network structure, they play a repeated game that consists of three decisions or stages:

1. Agents decide whether to cooperate or defect. A defecting agent obtains Temptation payoff (T = 3) and inflicts on each of the remaining agents in the population Hurt payoff (H = −1). If agents cooperate, no one's payoff is altered. Here we assume that the spillover cost of defection is global.
2. Agents observe other agents in their neighborhood who defected in stage 1 with a certain probability. For each observed defection, agents decide whether to punish the defector or not. Punishment is costly: one must pay Enforcement cost (E = −2) to impose Punishment cost (P = −9) on the defector. The opportunity to observe defection, and hence the possibility to punish it, is conditional on the existence of a link connecting defectors and punishers.
3. The third step includes the concept of metanorms: agents who fail to punish observed defection should be punished. Similar to the previous step, an agent who fails to punish an observed defection may not be caught. The probability of being seen not punishing a defecting agent given that defection is observed is the same as the probability of observing such defection. Network topology plays a critical role in this step: it determines who can see unpunished defection. Observing a defection requires links among the defector, un-punishing agent and metapunisher. A metapunisher pays Meta-enforcement cost (ME = −2) to meta-punish (MP = −9) an agent who decided not to punish a defector.

Parameters *boldness* and *vengefulness* characterize an agent's strategy. Boldness is an agent's propensity to defect, and determines the outcome of the first stage of the game. An agent that can defect will defect, if its boldness is greater than a random probability of being observed. Vengefulness is an agent's propensity to punish agents that it has observed defecting in the second stage of the game and to meta-punish agents that it has observed not punishing a defecting agent in the third. An agent punishes observed defectors or observed un-punishers with a probability equal to vengefulness. Following the original implementation by Axelrod, boldness and vengefulness are set as 3-bit strings denoting eight evenly distributed values from 0 to 1 (0/7, 1/7, …,7/7). Initial values of agents' boldness and vengefulness are determined randomly at the beginning of each simulation run and updated by an evolutionary mechanism.

The game is played four rounds called a generation. At the beginning of each generation, agents' payoffs are set to zero; at the end of a generation all payoffs for each round are accumulated and computed for each agent, and agents can change their strategies according to evolutionary forces of selection and mutation. We have adapted to local network structures a variant of selection mechanisms called roulette wheel in which the most successful agents in a given generation are the most likely to spread [81]: an agent picks a strategy played by other agents in its neighborhood with probability proportional to the other agents' fitness where an agent's fitness is equal to the difference between its payoff and the minimum payoff obtained in the neighborhood. Whenever an agent replicates a bitstring by invoking the selection mechanism, every bit of the bitstring has a certain probability of being flipped from 0 to 1 and vice versa called *mutation rate*. The game continues with a new generation playing with new agent strategies.

## Results

### Mathematical analysis

Given a specific network structure, the state of the game is a certain realization of agents' joint strategies, so the number of possible game states is 64^50^ corresponding to 64 strategies that any of the 50 players may choose. For any positive mutation rate, the model is an irreducible positive recurrent and ergodic discrete-time finite Markov chain [63], since the mutation operator guarantees the non-zero probability of transition from any state to any other state in one single step. This observation means that in the long run, the probability of finding the metanorms game in any of its states is non-zero and independent of the initial conditions of the game. This result guides our simulation experiments, because it guarantees that if we run simulations for long enough the limiting distribution approximates to the occupancy distribution.

The size of the state space of the game makes calculating the transition matrix of the Markov chain infeasible. We have to resort to other strategies to gain insights from a mathematical analysis of the model. In this section we propose a simpler mathematical abstraction of the metanorms game that is amenable to mathematical analysis and graphic visualization. This abstracted model suggests areas of stability and basins of attraction in the model and illustrates the expected dynamics of the metanorms graphically. We should stress that the conclusions of this analysis come from the simplified model, not the original one, so they must be verified by simulation.

Let's begin formalizing the model. Assume an undirected network 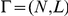 defined by a set of agents 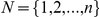
as nodes and a set of unweighted links among them 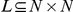. The payoff of agent *i* playing the metanorms game is defined by:
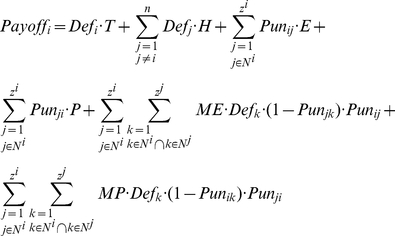
(1)


where *T*, *H*, *E*, *P, ME, MP* are the payoffs of the model, *n* is the number of agents, and 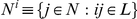
is the set of agents linked to any given 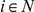. This set defines the *neighborhood* of *i*. 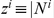 denotes the number of neighbors or degrees for agent *i*. Two indicator functions are also used:
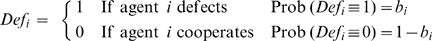


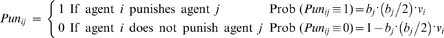



and vengefulness and boldness for each agent is denoted as *v_i_* and *b_i_*. Using vengefulness and boldness we calculate the expected payoff of agent *i* in one round as:
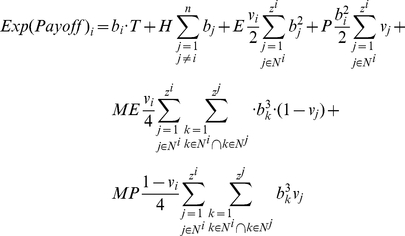
(2)


Eq. 2 depends on the exact realization of the network topology and exact strategies of each agent in the network. Let us now rewrite the Eq. 2 in terms of common statistics of the network topology.

First, let 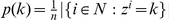 be the first-order degree distribution of network 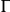. The clustering of an agent *i* with at least two neighbors is defined as:
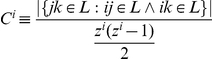



We define clustering coefficient for a degree in a given network as:
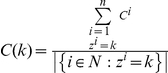



Assuming homogeneity in vengefulness and boldness 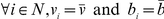 in the population, we can simplify the expected payoff of agent *i* as follows:
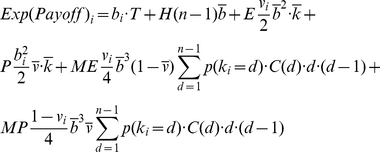
(3)


Eq. 3 expresses expected payoffs of homogeneous agents on a given network as a function of the first degree distribution, clustering distribution and the average degree 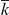 of the network. 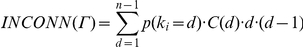 represents the average number of triplets per agent in network 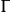. We call this statistic *interconnectedness* of network 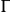. In other words, the dynamics and expected outcomes of the metanorms game may be highly influenced by the agents' average number of interactions and a certain measure of clustering of these interactions.

To characterize the long run outcomes of the game, we use the concept of *evolutionary stable state* (ESS) to identify the stability points of the game. This notion is inspired by the ideas proposed by Maynard Smith and Price [82] and developed by Weibull [83] and Colman [84]. An ESS in the metanorms game [53] is a state where:

- Every agent in the population Θ receives the same expected payoff, so evolutionary selection pressures will not lead the system away from the state,

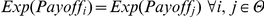

_._

- Any agent *m* that changes its strategy with *b_m_* as its new boldness and *v_m_* its new vengefulness, receives a strictly lower expected payoff than any other agent in the incumbent population *I* ≡ Θ-{*m*}, so if a single mutation occurs, the mutant agent will not be able to invade the population:

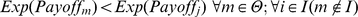
.

- Once a single agent *m* has changed its strategy, all other agents in the incumbent population *I* receive the same expected payoff, so a single mutant cannot distort the composition of the population except maybe by random drift:

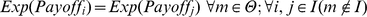
.

These three conditions above are enough to expect that any mutant will be removed from the game, providing strong restriction for stability in the dynamics of the model. If the system is not homogeneous, these conditions are not sufficient to guarantee in general that, if they are fulfilled in a certain state, the system will tend to revert to such a state after a single mutation. If the three conditions prevail in a certain state, we expect any mutant to be removed from the game, but the specific strategy among the incumbent population that will replace the mutant depends on the selection mechanism.

At this point we can establish two necessary conditions for a state to be evolutionary stable by assuming continuity in agent properties in Eq. 3. Let *m* be an arbitrary, but potentially mutant, agent with *b_m_* as its boldness and *v_m_* as its vengefulness in a given population of agents Θ. Let *I* be the set of incumbent agents in the population Θ excluding *m*. The following equation is a necessary condition for the population of agents to be in ESS. This condition can be easily grasped by realizing that if every agent has the same expected payoff as the necessary condition for ESS, and Eq. 4 does not hold for some agents *m* and *i*, the potentially mutant *m* can get a differential advantage over incumbent *i* by changing its boldness *b_m_*, meaning that the state under study cannot be evolutionary stable:



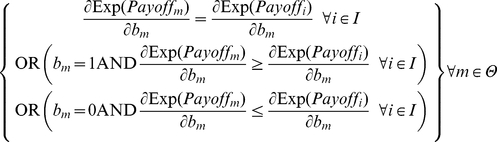
(4)


Similarly, we can obtain another necessary condition by substituting *v_m_* for *b_m_* in Eq. 4.
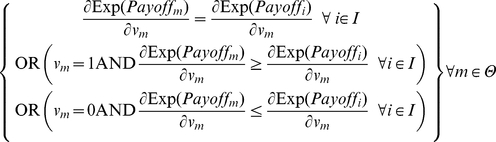
(5)


We can use Eq. 3 to evaluate the necessary derivatives:
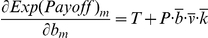





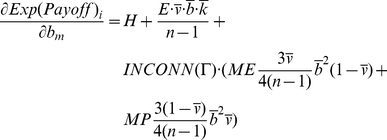
(6)

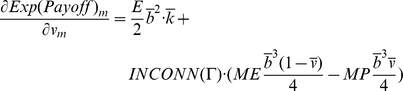


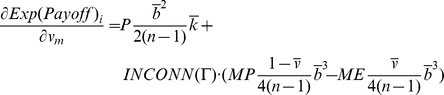



Generalizing the demonstration provided in [53], it can be proved that the system may have two different ESS, one where the norm collapses (*b_i_* = 1, *v_i_* = 0 for all *i*) and eventually another where the norm is established. This last ESS only appears depending on the relation between the average degree and the average number of triplets by agent, features that are determined by the network topology of the game.

**Figure 3:**
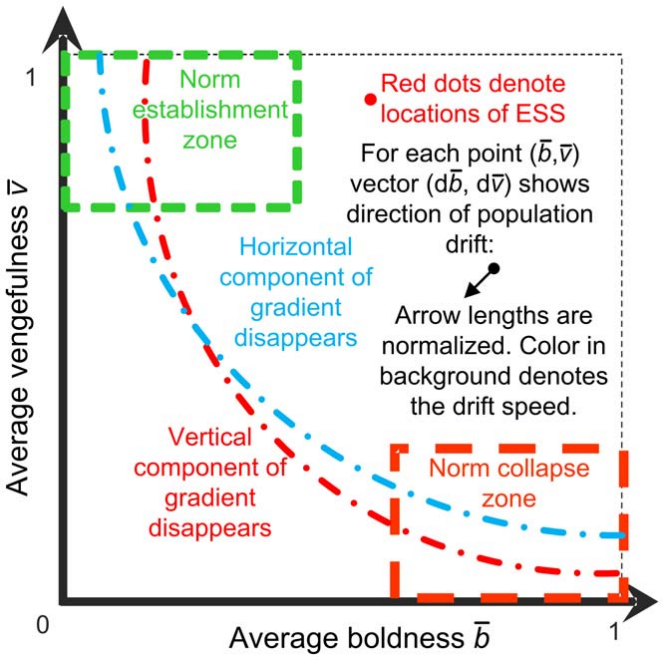
Legend for gradient maps. The map applies to both analytic and simulated gradient landscapes. The axes represent the average boldness and vengefulness of the population as its strategic characteristics. For each point, we measure the direction and speed of population drift. For analytical landscapes, we will also pinpoint the expected location of the evolutionary stable states. For simulation landscapes, we will be measuring the time that the simulation spends in each of the two key regions: norm emergence and norm collapse zones. Sample maps for different network topologies are shown in Figure 7.

**Figure 4:**
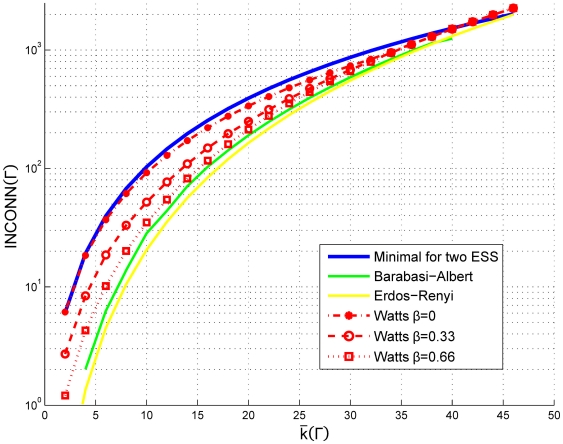
Minimal interconnectedness necessary for a cooperative evolutionary stable state. Minimal interconnectedness necessary for a cooperative evolutionary stable state to exist in the simplified analytical model for any given average degree of the network, compared to the expected interconnectedness of different network topologies with radius 1. Default metanorms parameters are assumed.

Evaluating gradients from Eq. 6 for any network topology and population characteristics leads to gradient maps of predicted population movements. The legend for these maps is described on Figure 3. For any constant value of average degree, the theoretical analysis suggests that the higher the average number of triplets, the more likely a cooperative ESS is to emerge and the bigger the size of its basin of attraction. On the contrary, for a constant average number of triplets, the higher average degree, the less likely a cooperative ESS is to emerge. Moreover, we can numerically calculate the minimum average number of triplets for each average degree in order to have an ESS in the area of norm establishment and compare it with different network topologies (Figure 4).

### Simulation

The results derived from the previous section are suggestive but we should keep in mind that they could have been obtained as consequences of simplifying assumptions not directly from the model explained in section two, since we abstracted the evolutionary mechanisms and the details of network topology, imposed continuity on agent properties, and worked only in terms of *expected* behavior. We need to verify if the suggested hypotheses in the equation-based approach can be generalized to the original metanorms game. Since that model, and especially when it is played on networks, is very complex, we have to resort to extensive simulation to gain insights on how it evolves. All simulations can be replicated with the source code of the model provided at http://josema.galan.name/models.

In the experiment designed to verify the behavior of the metanorms mechanism, we use the same payoff matrix, mutation rate and number of rounds per generation as in Axelrod's original paper (see Table 1).

**Table 1:** Summary of parameters used in the experiment.

Parameter	Value
Number of agents	50
Number of generations	50000
Mutation rate	0.01
Temptation payoff	*T* = 3
Hurt payoff	*H* = −1
Enforcement payoff	*E* = −2
Punishment payoff	*P* = −9
Meta-enforcement payoff	*ME* = −2
Meta-punishment payoff	*MP* = −9

**Figure 5:**
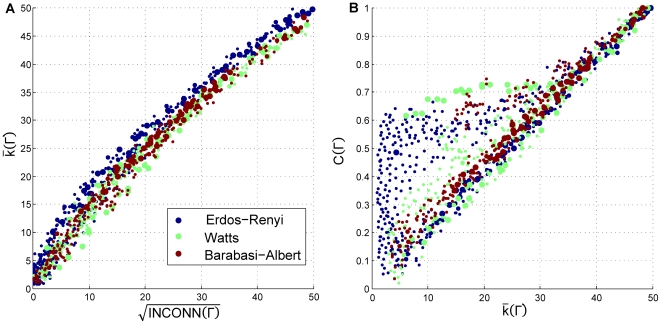
Distribution of tested data points obtained by sampling network topologies with radius 1 and 2. The distribution of the sampled network topologies, projected onto two dimensional views of key network statistics. The left panel shows sampled network topologies projected onto the square root of interconnectedness and the average degree of the network, while the right panel describes the sample density in the average degree and the clustering coefficient space.

We used the network generation algorithms mentioned in the previous section to create a sample of 6000 networks. The density of the sampled networks is plotted in Figure 5, projected onto the average degree against clustering coefficient of the network and the square root of the interconnectedness spaces. Note that clustering, interconnectedness and the average degree are not independent variables.

The results obtained from simulations allow us to perform two types of analyses, an analysis of the long-run limiting behavior of metanorms, and an analysis of the dynamics to compare with the expected dynamics predicted by the simplified theoretical model.

To study the influence of the network topology on the long-run behavior of the model, recall that when mutation rate is greater than zero, the metanorms game on networks is a time-homogeneous Markov chain (THMC) in which the limiting distribution coincides with the occupancy distribution as the long-run fraction of the time that the THMC spends in each state. Therefore, we can approximate the limiting distribution by computing the frequency of simulation in each state. We have defined the following zones:

- Norm Collapse: the simulation is in states where the average boldness is at least 6/7 and the average vengefulness is no more than 1/7.
- Norm Establishment: the simulation is in states where average boldness is no more than 1/7 and average vengefulness is at least 6/7.

**Figure 6:**
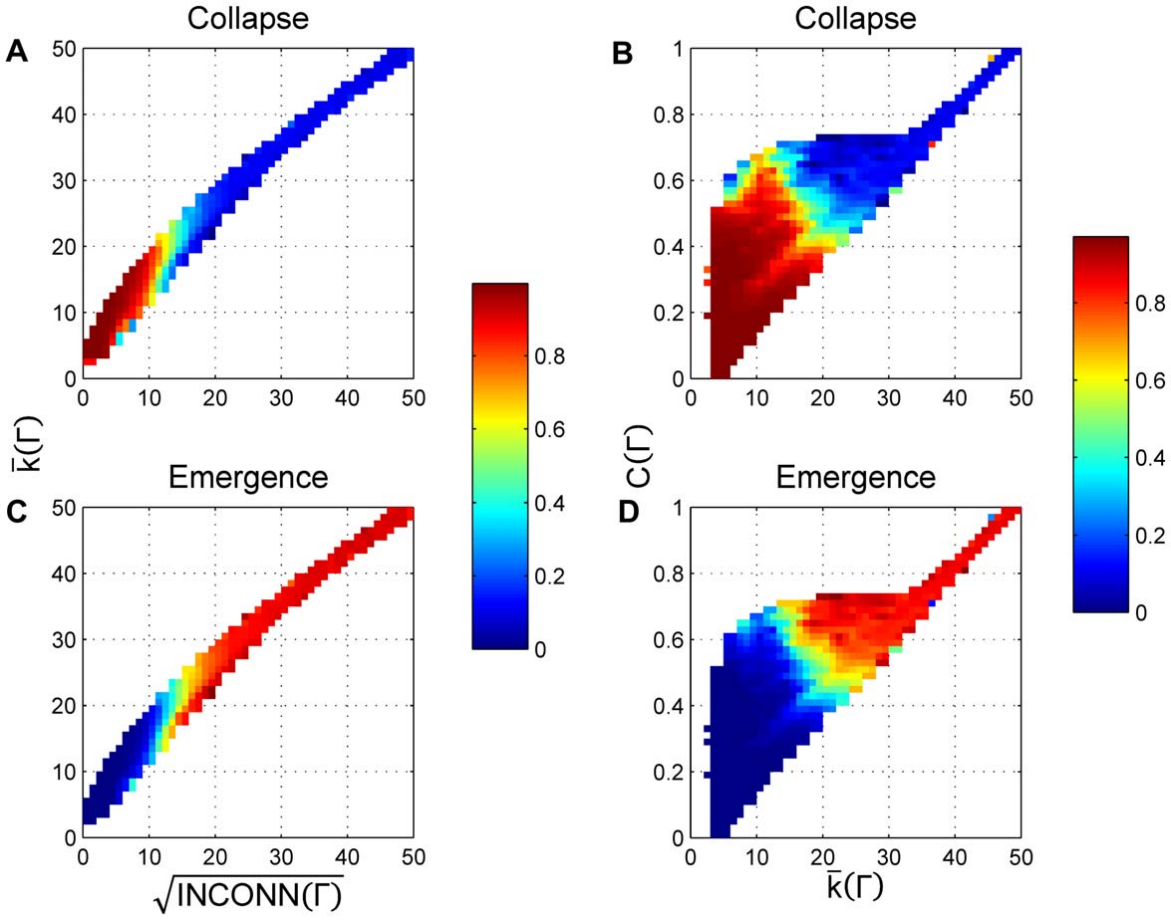
Proportion of time spent in the emergence and collapse zones. Proportion of time that the simulation spends in the norm collapse and emergence zones as a function of key network statistics using similar projections as those in Figure 5. Color codes the fraction of simulation time spent in each zone computed for each bin. Time spent outside either zone is insignificant.

We have computed the time that a single simulation is in either zone. In Figure 6 we have measured the long-run fraction of time, averaged over runs and over network topologies that the simulation is in cooperative norm emergence and collapse zones as a function of average degree and root square of interconnectedness and as function of average degree and clustering coefficient of the network. First, these results suggest that the ESS obtained analytically in the simplified model are in fact the only ESS in the system since the time that the simulation is out of these two zones is not significant.

Second, we observe that on average the influence of average degree, the number of triplets and clustering coefficient behave as predicted analytically. The higher the average number of triplets the more time the simulation spends in norm establishment zone; on the contrary, for a given average number of triplets, the higher average degree of the network, the lower the probability of finding the simulation in the norm establishment zone. These results suggest that an important part of the limiting behavior of the game can be explained by two simple statistics of the network topology.

Last, we have found some variance in the results. For example, we do find the norm establishment in sparser networks that are analytically predicted not to reach the norm establishment zone. This indicates that agent and network heterogeneity, for example local “clumps” denser than the whole network by chance, and the specifics of the evolutionary mechanism also play important roles in norm establishment as they may allow for “seeding” the cooperative norm in the network [67], [85], [86].

**Figure 7:**
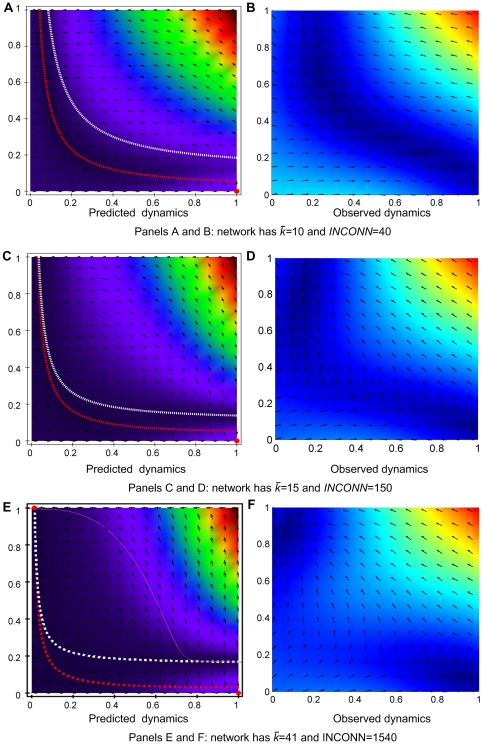
Predicted versus observed dynamics of the metanorms game for three networks. For simulated results, the mutation rate was set to 0.01. Color codes the speeds of movement of the population, either computed analytically or measured from the simulation with blue being the slowest and red the fastest. Figure 3 contains the legend for the graphs. In panel B, simulated population spends 95% of time in norm collapse zone. In panel D, the proportion of time in the norm collapse zone drops to 50%. In panel E, the simulation spends 95% of time in the norm emergence zone and 5% in the norm collapse zone.

We have also analyzed data from simulation to determine the match between gradient maps obtained in the simplified mathematical analysis and simulation model dynamics. The first column of Figure 7 represents the mathematically predicted dynamics, whereas the second column presents simulated speed and direction of population drift. We observe that in terms of speed and direction of trajectories the predicted dynamics match, especially for those cases where there is only one clear ESS or two ESSs. The interesting result appears when there is only one predicted ESS but we are close to having another in the zone of norm establishment. The observed dynamics show possible quasi-stable states in norm emergence earlier than predicted by the equation approach. This effect may be the consequence of mutation rate and variance of selection mechanism that make it difficult to escape from the norm establishment zone [53].

## Discussion

We have adapted the theoretical model of metanorms to guide agent interactions on static networks. Our analytical and computational results show that the interaction structure influences the effectiveness of the metanorms mechanism. In particular, we identified the average degree, clustering coefficient and interconnectedness as the average number of triplets per agent as key aspects that contribute to sustaining or collapsing norms of cooperation in networked populations. Higher clustering coefficient and average number of triplets increases cooperative behavior, suggesting that translating bilateral to trilateral interaction promotes cooperation. Comparing the results of our simplified mathematical analysis with those of computational modeling, we have also shown that some evolutionary details influence in the model dynamics that stabilize the zone of norm establishment.

The networks used for the analysis have been numerous and diverse, nevertheless we have not analyzed all possible configurations. Some recent studies [67], [85]–[87] have proved that community structure [88], subsets of nodes that are relatively densely connected to each other but sparsely connected to other dense groups, can be also an important parameter in the behavior of games in networks, although the network generators used in our analysis are not particularly designed to take into account this effect. Further research may clarify the effect of more complex topologies, particular evolutionary details or scale on the effectiveness of metanorms as mechanism to sustain cooperation in social dilemmas.
